# Obstetric hemorrhage risk assessment tool predicts composite maternal morbidity

**DOI:** 10.1038/s41598-021-93413-3

**Published:** 2021-07-19

**Authors:** Emer L. Colalillo, Andrew D. Sparks, Jaclyn M. Phillips, Chinelo L. Onyilofor, Homa K. Ahmadzia

**Affiliations:** 1 Department of Obstetrics and Gynecology, St. Luke’s University Health Network, Allentown, PA USA; 2 Department of Surgery, George Washington University School of Medicine & Health Sciences, Washington, DC, USA; 3 Division of Maternal Fetal Medicine, Department of Obstetrics, Gynecology and Reproductive Sciences, University of Pittsburgh School of Medicine, Pittsburgh, PA USA; 4 Division of Maternal Fetal Medicine, Department of Obstetrics and Gynecology, George Washington University School of Medicine & Health Sciences, Washington, DC, 20037 USA

**Keywords:** Risk factors, Translational research, Comorbidities, Reproductive signs and symptoms

## Abstract

Obstetric hemorrhage is one of the leading preventable causes of maternal mortality in the United States. Although hemorrhage risk-prediction models exist, there remains a gap in literature describing if these risk-prediction tools can identify composite maternal morbidity. We investigate how well an established obstetric hemorrhage risk-assessment tool predicts composite hemorrhage-associated morbidity. We conducted a retrospective cohort analysis of a multicenter database including women admitted to Labor and Delivery from 2016 to 2018, at centers implementing the Association of Women’s Health, Obstetric, and Neonatal Nurses risk assessment tool on admission. A composite morbidity score incorporated factors including obstetric hemorrhage (estimated blood loss ≥ 1000 mL), blood transfusion, or ICU admission. Out of 56,903 women, 14,803 (26%) were categorized as low-risk, 26,163 (46%) as medium-risk and 15,937 (28%) as high-risk for obstetric hemorrhage. Composite morbidity occurred at a rate of 2.2%, 8.0% and 11.9% within these groups, respectively. Medium- and high-risk groups had an increased combined risk of composite morbidity (diagnostic OR 4.58; 4.09–5.13) compared to the low-risk group. This established hemorrhage risk-assessment tool predicts clinically-relevant composite morbidity. Future randomized trials in obstetric hemorrhage can incorporate these tools for screening patients at highest risk for composite morbidity.

## Introduction

Obstetric hemorrhage is the leading cause of maternal mortality worldwide^1, 2^. Furthermore, recent data has shown that perinatal hemorrhage has steadily increased in developed countries, including the United States^3–8^. Subsequently, there has been a drive to improve identification of patients at risk for hemorrhage through risk-stratification tools and targeted labor and delivery protocols^9, 10^.

Although there are numerous hemorrhage risk-prediction models in use, there remains a gap in literature describing the ability of these risk-prediction tools to identify hemorrhage-related maternal morbidity^11–14^. The Association of Women’s Health, Obstetric and Neonatal Nurses (AWHONN) created a hemorrhage risk-prediction tool that classifies women as low-, medium-, or high-risk for hemorrhage, to be implemented upon admission to labor and delivery, pre-birth, and immediately postpartum^15, 16^. Studies suggest that the AWHONN tool is easily implemented, with moderate sensitivity for identifying women who are at risk for severe postpartum hemorrhage^16, 17^. This risk assessment structure is used and cited by the American College of Obstetricians and Gynecologists (ACOG) Safe Motherhood Initiative, and is implemented at a national level^18, 19^. However, like many other hemorrhage risk-assessment tools, little has been studied regarding how this tool specifically predicts the morbidity associated with obstetric hemorrhage^11, 17^.

Recently, studies in this field have begun to utilize a composite maternal morbidity outcome to assess the efficacy of hemorrhage interventions^20–24^. To date, however, there are no studies utilizing a risk-assessment tool to predict hemorrhage-related maternal morbidity, as measured by a composite score. Our study aims to assess the extent to which an established hemorrhage risk-assessment tool predicts composite obstetric hemorrhage-associated morbidity. By evaluating composite hemorrhage-associated morbidity specifically, we aim to describe the association between hemorrhage risk score and poor maternal outcomes. Although there is no standard definition for hemorrhage-associated morbidity, we utilized a definition containing blood loss greater than one liter, blood transfusion, intensive care unit (ICU) admission, need for hemorrhage-related procedures (hysterectomy, dilation and curettage). Understanding the relationship between hemorrhage-risk score and hemorrhage-associated morbidity may be critical in identifying patient populations who could benefit from additional risk-reducing interventions at the time of delivery, such as tranexamic acid.

## Materials and methods

This is a retrospective study conducted using data from a multicenter database that included women who were admitted to Labor and Delivery (L&D) from June 2016 to June 2018. Data are pulled from the electronic medical record and includes clinical- and laboratory-based variables. Nineteen hospitals were included in the database. Three hospitals had an average annual delivery volume less than 500 (16%), seven had 500–999 (37%), seven had 1000–2999 (37%) and two were greater than 3000 (11%). The geographic distribution of the hospitals were East coast (N = 5; 26%), Central (N = 7; 37%), and West coast (N = 7; 37%).

All centers involved in our study implemented universal screening using the hemorrhage risk-assessment tool on June 1, 2016. This electronic tool via Cerner platform was administered by a labor and delivery nurse at the time of admission (in addition to pre-birth and immediately postpartum), after which a risk score was automatically generated and documented in the patient electronic medical record. This tool categorized all patients as low-, medium- or high-risk for hemorrhage, using well-established risk factors for hemorrhage (Table 1). Analyses were performed on medium, high, and a combined medium and high risk group.

**Table 1:** AWHONN hemorrhage risk assessment^29^.

Low risk	Medium risk*	High risk
No previous uterine incision ≤ 4 Previous vaginal births No known bleeding disorder No history of PPH Singleton pregnancy	Induction of labor > 4 Prior vaginal births Prior cesarean birth or prior uterine incision Large uterine fibroids History of one previous PPH Chorioamnionitis Fetal demise Morbid obesity (BMI > 35) Estimated fetal weight > 4 kg Family history in first degree relative who experienced PPH Polyhydraminos	Active bleeding more than bloody show Suspected accreta or percreta Placenta previa, low lying placenta Known coagulopathy History of more than one previous PPH Hematocrit < 30 and other risk factors Platelets < 100 k

*AWHONN* Association of Women’s Health, Obstetric and Neonatal Nurses, *PPH* Postpartum hemorrhage, *BMI* body mass index.

*If two or more medium risk items are found then that classifies as 'high risk'.

Any patient who was admitted for delivery met criteria for this study. Estimated blood loss (EBL) was inputted by trained L&D nurses into electronic medical records with input from the delivering physician. Patients were excluded if they had no data available for hemorrhage risk assessment. Hemorrhage risk scores were electronically abstracted with the aid of a hospital information technology official and a labor and delivery nurse. Through the Office of Human Research, The George Washington University Institutional Review Boards (IRBs) approved protocols for study procedures (IRB 180249 on 12/18/2018 and IRB 061611 on 7/27/2017). All data were fully anonymized prior to accessing the information for the study and the George Washington University IRBs waived requirement of informed consent for this retrospective analysis. In addition, all methods performed in this study were done in accordance to the guidelines and regulations of The George Washington University IRB.

Maternal demographic data were collected including maternal medical and surgical history and obstetric data. Data from the hemorrhage risk-assessment tool were extracted from the database. Outcomes related to EBL ≥ 1000 mL, blood transfusion, ICU admission, were evaluated in each risk group. Blood transfusion was defined as administration of any amount of packed red blood cell (pRBCs) during the intrapartum or postpartum time course.

### Statistical analysis

Patient variables between low-, medium-, and high-risk postpartum hemorrhage cohorts defined from the database were compared using Chi-square for categorical variables, independent samples t-test and one-way ANOVA for parametric continuous variables, and Mann–Whitney U/Wilcoxon rank-sum test for nonparametric continuous variables.

A composite outcome incorporating obstetric hemorrhage (defined as EBL ≥ 1000 mL by gravimetric method), blood transfusion, ICU admission, and/or additional hemorrhage-related complications (hysterectomy, dilation and curettage) was then compared between the low-, medium-, and high-risk postpartum hemorrhage cohorts. Respective incidences of composite outcome and each individual component by hemorrhage cohort were reported. Statistical diagnostics including sensitivity, specificity, positive predictive value (PPV), negative predictive value (NPV) and diagnostic odds ratio (dOR = relative risk/RR) were calculated for the composite hemorrhage-related morbidity outcome between medium- and low-risk cohorts, high- and low-risk cohorts, and combined medium- plus high-risk relative to low-risk hemorrhage cohorts.

All statistical analysis was performed using SAS version 9.4 (SAS Institute Inc., Cary, NC) and R version 3.6.2 (R Foundation for Statistical Computing, Vienna, Austria). A two-sided p-value less than 0.05 was considered statistically significant.

### Ethics approval

IRB approval through George Washington University, waiver of consent given retrospective nature.

## Results

In this cohort of 56,903 women, 14,803 (26%) were categorized as low-risk, 26,163 (46%) as medium-risk and 15,937 (28%) as high-risk of obstetric hemorrhage. Maternal characteristics, by cohort, are summarized in Table 2. Increasing risk cohort was associated with multiple variables not used in stratification. Notably, older age, higher BMI, rate of cesarean delivery, number of prior cesareans, and preeclampsia were all associated with categorization in an increased risk cohort (all respective p < 0.0001; Table 2). Obstetric hemorrhage occurred at a rate of 2.1%, 7.6% and 11.4% within the low-, medium- and high-risk groups, respectively. Composite maternal morbidity was found to occur in 2.2% of the cases characterized as low-risk, 8.0% of medium-risk and 11.9% of high-risk (Table 3). We observed that blood transfusion, ICU admission, and/or additional hemorrhage-related complications (hysterectomy, dilation and curettage) occurred at a frequency of 0.1% 0.4% and 0.5% over the respective hemorrhage incidences (defined as blood loss of 1000 cc or greater) of 2.1%, 7.6% and 11.4%, respectively.

**Table 2:** Patient Variables by PPH risk assessment category (overall cohort, N = 56,903).

Patient variable	Low (n = 14,803)	Medium (n = 26,163)	High (n = 15,937)	Overall *P*	Medium vs low *P*	High vs low *P*
Maternal age	27.5 ± 6.3	28.4 ± 6.5	29.1 ± 6.8	< 0.0001	< 0.0001	< 0.0001
Delivery BMI	29.4 ± 5.9	31.7 ± 6.9	34.6 ± 8.1	< 0.0001	< 0.0001	< 0.0001
**Delivery mode**				< 0.0001	< 0.0001	< 0.0001
CD*	1214 (8.2)	12,735 (48.7)	7326 (46.0)			
Vaginal	13,541 (91.5)	13,240 (50.6)	8439 (53.0)			
VBAC*	48 (0.3)	188 (0.7)	172 (1.1)			
**Race**				< 0.0001	< 0.0001	< 0.0001
Black	2374 (16.0)	4060 (15.5)	3454 (21.7)			
White	7453 (50.4)	14,143 (54.1)	7811 (49.0)			
Asian	712 (4.8)	1095 (4.2)	703 (4.4)			
Other/Unk	4264 (28.8)	6865 (26.2)	3969 (24.9)			
**Insurance**				< 0.0001	< 0.0001	< 0.0001
Private	8317 (56.2)	14,048 (53.7)	9195 (57.7)			
Medicaid	4931 (33.3)	8834 (33.8)	4925 (30.9)			
Self-pay	401 (2.7)	954 (3.7)	282 (1.8)			
Other/Unk*	1154 (7.8)	2327 (8.9)	1535 (9.6)			
**Marital status**				0.0024	0.0004	0.0140
Married	7079 (47.8)	12,897 (49.3)	7830 (49.1)			
Single	6969 (47.1)	11,809 (45.1)	7244 (45.5)			
Other/Unk	755 (5.1)	1457 (5.6)	863 (5.4)			
Gestational diabetes	550 (3.7)	1784 (6.8)	1466 (9.2)	< 0.0001	< 0.0001	< 0.0001
Pre-gestational diabetes	72 (0.5)	324 (1.2)	310 (2.0)	< 0.0001	< 0.0001	< 0.0001
Chronic hypertension	76 (0.5)	399 (1.5)	446 (2.8)	< 0.0001	< 0.0001	< 0.0001
Smoke during	623 (4.2)	1249 (4.8)	810 (5.1)	0.0012	0.0085	0.0003
Preeclampsia	127 (0.9)	688 (2.6)	647 (4.1)	< 0.0001	< 0.0001	< 0.0001
Inter-pregnancy interval < 1 year	237 (1.6)	591 (2.3)	394 (2.5)	< 0.0001	< 0.0001	< 0.0001
Placenta previa	6 (< 0.1)	51 (0.2)	142 (0.9)	< 0.0001	< 0.0001	< 0.0001
DIC	–	2 (< 0.1)	19 (0.1)	< 0.0001	0.5384	< 0.0001
History of PPH*	125 (0.8)	608 (2.3)	1162 (7.3)	< 0.0001	< 0.0001	< 0.0001
Dinoprostone	477 (3.2)	2398 (9.2)	1911 (12.0)	< 0.0001	< 0.0001	< 0.0001
Misoprostol	1094 (7.4)	3550 (13.6)	2821 (17.7)	< 0.0001	< 0.0001	< 0.0001
Oxytocin	12,174 (82.2)	23,693 (90.6)	14,453 (90.7)	< 0.0001	< 0.0001	< 0.0001
Use of antibiotics	6312 (42.6)	16,472 (63.0)	10,455 (65.6)	< 0.0001	< 0.0001	< 0.0001
Vacuum	434 (2.9)	1603 (6.1)	1163 (7.3)	< 0.0001	< 0.0001	< 0.0001
Forceps	92 (0.6)	212 (0.8)	257 (1.6)	< 0.0001	0.0324	< 0.0001
Eclampsia	15 (0.1)	61 (0.2)	50 (0.3)	0.0003	0.0029	< 0.0001
HELLP*	3 (< 0.1)	15 (< 0.1)	10 (< 0.1)	0.1767	0.0855	0.0703
**Number of previous CD**				< 0.0001	< 0.0001	< 0.0001
0	14,697 (99.3)	23,032 (88.0)	14,164 (88.9)			
1	73 (0.5)	1903 (7.3)	1033 (6.5)			
2+	33 (0.2)	1228 (4.7)	740 (4.6)			
Gestational age < 39 weeks	5793 (39.1)	9705 (37.1)	6393 (40.1)	< 0.0001	< 0.0001	0.0792
Intrapartum abruption	57 (0.4)	107 (0.4)	291 (1.8)	< 0.0001	0.7127	< 0.0001
Placenta accreta	–	3 (< 0.1)	38 (0.2)	< 0.0001	0.5578	< 0.0001
Intrapartum bleeding	3 (< 0.1)	11(< 0.1)	21 (0.1)	< 0.0001	0.2519	0.0005
Platelets < 150,000	2039 (13.8)	3793 (14.5)	2134 (13.4)	0.0042	0.0441	0.3260
Hematocrit < 32%	2735 (18.5)	5751 (22.0)	3418 (21.5)	< 0.0001	< 0.0001	< 0.0001
**Parity**				< 0.0001	< 0.0001	< 0.0001
0	3217 (21.7)	5023 (19.2)	3525 (22.1)			
1	7095 (47.9)	12,328 (47.1)	7344 (46.1)			
2	2322 (15.7)	4328 (16.2)	2260 (14.2)			
3	1411 (9.5)	2790 (10.7)	1512 (9.5)			
4+	758 (5.1)	1784 (6.8)	1296 (8.1)			
Multiple gestation	33 (0.2)	718 (2.7)	911 (5.7)	< 0.0001	< 0.0001	< 0.0001

*Risk factors: BMI* body mass index, *CD* cesarean delivery, *VBAC* vaginal birth after cesarean, *Unk* unknown, *DIC* disseminated intravascular coagulation, *PPH* postpartum hemorrhage, *HELLP* hemolysis, elevated liver enzymes, low platelets.

**Table 3:** Frequency of composite morbidity and individual components to composite across risk groups.

	Low (n = 14,803)	Medium (n = 26,163)	High (n = 15,937)	Medium + high (n = 42,100)
Composite morbidity	330 (2.2)	2084 (8.0)	1898 (11.9)	3982 (9.5)
Obstetric hemorrhage	304 (2.1)	1986 (7.6)	1822 (11.4)	3808 (9.1)
Blood transfusion	26 (0.2)	109 (0.4)	123 (0.8)	232 (0.6)
ICU admission	13 (0.1)	45 (0.2)	41 (0.3)	86 (0.2)
Additional complication	–	2 (< 0.1)	23 (0.1)	25 (< 0.1)

We observed a significantly increased risk of composite morbidity for both the medium- (dOR 3.80; 95% CI 3.37–4.27) and the high-risk group (dOR 5.93; 5.26–6.68) when compared individually to the low-risk group. Importantly, those in the combined medium- and high-risk groups were found to have a 4.58 times higher odds of experiencing a composite morbidity than the low-risk group (p < 0.0001; Table 4). When comparing the combined medium- and high-risk groups to the low-risk group, NPV for composite morbidity was 0.98 and positive predictive value (PPV) was 0.10 (Table 4).

**Table 4:** Statistical diagnostics of hemorrhage risk-assessment tool for prediction of composite maternal morbidity.

Diagnostic	Medium vs low risk	High vs low risk	Medium + high vs low risk
Sensitivity	0.86	0.85	0.92
Specificity	0.38	0.51	0.28
PPV	0.08	0.12	0.10
NPV	0.98	0.98	0.98
Diagnostic OR (95% CI)	3.80 (3.37–4.27)	5.93 (5.26–6.68)	4.58 (4.09–5.13)

## Discussion

These findings provide insight into the ability of an established hemorrhage risk-assessment tool to predict hemorrhage-related morbidity. This study demonstrates a significantly increased risk of composite hemorrhage-related morbidity for patients categorized as both medium and high risk for hemorrhage, when compared to the low risk group.

In the United States, the overall rate of severe maternal morbidity (SMM) has increased over two-hundred percent since 1993, driven primarily by an increased utilization of blood transfusion^25^. Obstetric hemorrhage remains one of the leading causes of SMM and accounts for over half of all identified SMM events^1^. One of the most common preventable factors in SMM is failure to identify patients who are at high risk for obstetric complications, which can significantly delay diagnosis, management, and treatment^26^. In response, risk assessment programs and obstetric care bundles have been implemented on a national level to better identify patients who are at the highest risk for obstetric hemorrhage^19, 27–32^.

The AWHONN risk assessment tool has emerged as a simple, easy to implement, and low-cost hemorrhage-risk prediction tool that is now widely accepted across the United States. It was designed as a risk-stratification method, identifying patients with low, medium, or high risk for obstetric hemorrhage^29^. Prior studies have suggested that this tool works moderately well to identify patients at highest risk for both obstetric hemorrhage and transfusion^17^, and when implemented, may reduce rates of maternal morbidity^33^. Our study demonstrates that, in addition to predicting obstetric hemorrhage, the AWHONN risk assessment tool identifies women at highest risk for hemorrhage-related morbidity, which may be more clinically relevant. However, it is key to note that within this study, morbidity was primarily driven by PPH. With an excellent diagnostic odds ratio (OR), those categorized as high or medium risk by the AWHONN tool can receive extra attention in preparation for hemorrhage and hemorrhage-associated morbidities. Additionally, with an NPV value of 0.98, this admission questionnaire functions well as a screening test for hemorrhage-related morbidity, reliably separating those who are at low risk, as 98% of those classified as low-risk did not experience a hemorrhage-related morbidity. Though our PPV indicates that a patient classified as medium- or high-risk only has a 9% chance of experiencing a hemorrhage-related morbidity—due to a somewhat rare outcome—this follows with general guidelines for a screening test, with a high NPV of 0.98 and low risk for categorization of patients in a higher risk group. Importantly, this sheds light on the poor diagnostic accuracy of these tools at predicting either hemorrhage or hemorrhage-associated morbidity and sheds light on the need for a more accurate risk assessment tool.

Severe maternal morbidity and mortality remain important public health concerns, affecting 2.9/1000 births, with the majority of morbidity attributed to obstetric hemorrhage^1^. Understanding the ability of existing hemorrhage risk-prediction tools to predict hemorrhage-related morbidity will increase awareness of a patient’s high-risk status, encourage early management of hemorrhage, and potentially allow healthcare providers to selectively implement preventative hemorrhage interventions (e.g., active management of the third stage of labor). Further understanding of how this tool predicts those at highest risk for morbidity would also improve our ability to describe and study high-risk patient populations. Importantly, data from this tool should be abundant, as it is already in use on a national level, and is easy to implement and track. Especially in health centers with low levels of maternal care, this tool has the potential to efficiently allocate more resources to monitor those who have an increased risk of hemorrhage. Lastly, clinically relevant composite hemorrhage morbidity and baseline rates are essential to design randomized clinical trials assessing interventions to reduce obstetric hemorrhage.

Strengths of this study include the large number of patients and the use of a multicenter database to capture a diverse maternal population. Our dataset is contemporary, collected from 2016 to 2018, making it generalizable to our modern patient population. The retrospective design of our study poses several limitations, including the loss of complete data, which may have disproportionately affected more complicated, and therefore more hectic, deliveries, where data may not have been entered in entirety. Additionally, hemorrhage risk scores were calculated for each patient at the time of admission to labor and delivery in a prospective manner. An important limitation to our study includes potential error in hemorrhage risk score calculation. Patients who were classified as low risk were identified to have risk factors that would place them in a medium or high risk category. This error is either an error in score calculation or patient demographic data available within our database and is a limitation of our retrospective analysis. This might lead to incorrect charting of bedside procedures such as bedside curettage or uterine tamponade device placement. Use of EBL may lead to incorrect categorization of hemorrhage status; however, since this misclassification bias was across all groups this is something that likely did not impact our findings. As this study was retrospective and across multiple clinical sites, technique for measuring EBL was not standardized between hospitals or providers. In addition, it is possible that transfusion may have occurred before delivery as time of transfusion was not documented as a variable thus, potentially changing the risk stratification of participants. Additionally, data were collected regarding ICU admission, however there was no ability to discern the diagnosis leading to admission. It is possible that there was misclassification of hemorrhage risk categories upon admission (for example, a patient with a prior cesarean was mistakenly categorized as low risk) because the hemorrhage risk scores calculated by nurses on labor and delivery and not verified by a physician. Furthermore, there is no universal definition of hemorrhage-related maternal morbidity, which limits the generalizability of our study in comparison to other works, as well as national and global statistics, which may have used other definitions. Lastly, the AWHONN tool recommends that periodic risk assessments should be conducted by facilities that choose to use this tool. Currently, there is no standardized way to evaluate this tool thus its effectiveness may differ by location.

In conclusion, our study demonstrates that the AWOHNN hemorrhage risk-prediction tool not only identifies patients at highest risk for obstetric hemorrhage, but also can be used as a screening tool for those at risk for hemorrhage-related morbidity. Women who were categorized as being at high risk for obstetric hemorrhage using the AWHONN hemorrhage risk assessment tool were six times more likely to experience hemorrhage-related morbidity compared to those who were low-risk. One setback of this tool is the low PPVs indicate a high false positive rate thus, there needs to be additional research done to develop better tools to optimize prediction of obstetric hemorrhage risk. Furthermore, morbidity was primarily driven by PPH, hence the morbidity outcome among the different groups were similar. Thus, further studies looking to assess the accuracy of prediction tools can consider using PPH as the primary way to compare different risk categories.

## Data Availability

Data will not be available for direct download or analysis given not included in original IRB submission Of note, findings were presented at the 40th Annual SMFM Meeting, February 3-8, 2020; Grapevine, TX.
